# Levels of myeloid-derived suppressor cell-like cells in early sepsis: a comparative study with non-septic patients

**DOI:** 10.3389/fimmu.2026.1763005

**Published:** 2026-03-31

**Authors:** Lijing Jia, Huawei Wang, Ling Long, Chen Ge, Ze Zhang, Hua Chen, Jing Wang, Heling Zhao

**Affiliations:** 1 Hebei Medical University, Shijiazhuang, China; 2 High Dependency Unit, Hebei General Hospital, Shijiazhuang, China; 3 Department of Intensive Care Medicine, Hebei General Hospital, Shijiazhuang, China

**Keywords:** arginase-1, inducible nitric oxide synthase, MDSC-like cells, myeloid-derived suppressor cells, sepsis

## Abstract

**Background:**

Myeloid-derived suppressor cells (MDSCs) are immature myeloid cells with immunosuppressive functions. While early expansion of MDSCs may be protective in various pathological states, their accumulation and role might differ in sepsis. This study aimed to compare the differences in circulating myeloid cells with MDSC phenotypes and their subsets between septic and non-septic patients in the early stage.

**Methods:**

This was a prospective, single-center, observational cohort study. Critically ill patients were enrolled and divided into sepsis and non-sepsis groups. Flow cytometry was used to determine the percentages of peripheral blood CD11b^+^CD33^+^HLA-DR^−^/low cells (herein referred to as MDSC-like cells) and their subsets (M-MDSC-like and PMN-MDSC-like cells). Levels of arginase-1 (ARG-1) and inducible nitric oxide synthase (iNOS) were measured. Clinical data were collected. All patients were followed for 28 days to record mortality.

**Results:**

Sixty patients were enrolled (sepsis group: n=38; non-sepsis group: n=22). No significant differences were found in gender, age, APACHE II score, ICU length of stay, or 28-day mortality between the two groups. However, the Charlson Comorbidity Index (CCI) was higher in the sepsis group (P = 0.005). Compared to the non-sepsis group, septic patients had significantly lower percentages of total MDSC-like cells and M-MDSC-like cells (P = 0.006; P = 0.003), while PMN-MDSC-like cells showed no difference. ARG-1 levels were higher in the sepsis group (P = 0.030). Furthermore, the sepsis group exhibited significantly elevated levels of IL-6, CRP, PCT, and SOFA scores (P<0.05), lower lymphocyte counts (P = 0.017), and more pronounced coagulation abnormalities, hypoalbuminemia, and increased cardiac/renal markers. Within the sepsis group, non-survivors had a significantly higher percentage of PMN-MDSC-like cells than survivors (P = 0.012).

**Conclusion:**

In the early stage, septic patients exhibit a distinct response profile of myeloid cells with MDSC phenotypes compared to non-septic patients, characterized by attenuated expansion of total MDSC-like cells and M-MDSC-like cells but enhanced ARG-1 expression, alongside more severe inflammation, organ dysfunction, and lymphopenia. An elevated percentage of PMN-MDSC-like cells is associated with poor prognosis in sepsis.

## Introduction

Myeloid-derived suppressor cells (MDSCs) are a group of innate immune cells with immunosuppressive properties present in the human body. Under physiological conditions, their numbers are very low, with only a small fraction of immature myeloid cells migrating from the bone marrow to the periphery before completing differentiation. Immature myeloid cells account for approximately 0.5% of peripheral blood immune cells in healthy individuals (1, 2), while normally generated MDSCs constitute less than 1% of neutrophils (3). However, under pathological conditions, they undergo rapid and substantial expansion, migrate, and are recruited to specific sites, where they exert their physiological and pathological functions and influence patient prognosis (4–6). MDSCs were formally named in 2007 (7) and have attracted particular attention due to their role in inducing systemic and local immunosuppression and promoting the recruitment of other immunosuppressive cells (8).

MDSCs are currently classified into two principal subtypes based on morphological and phenotypic criteria: polymorphonuclear MDSCs (PMN-MDSCs) resembling granulocytes, and monocytic MDSCs (M-MDSCs) sharing features with monocytes (9). Immunophenotyping reveals distinct surface marker profiles: human PMN-MDSCs are characterized by CD33+CD11b+CD15+HLA-DRlow expression, while M-MDSCs exhibit CD33+CD11b+CD14+HLA-DRlow markers (10). Additionally, researchers have identified a minor population (<5% of total MDSCs) of developmentally primitive cells designated as early-stage MDSCs (e-MDSCs) (8). These precursor cells emerge during initial disease progression and display a unique CD33+CD11b+HLA-DRlowCD14-CD15- profile, lacking both granulocytic and monocytic differentiation markers (11).

While the role of MDSCs in tumor immunology has been well characterized, their functions in other fields remain less explored. Sepsis is defined as life-threatening organ dysfunction caused by a dysregulated host response to infection (12). Septic shock, a subset of sepsis, is associated with severe circulatory, cellular, and metabolic abnormalities, leading to a significantly higher mortality risk compared to sepsis alone (13). At its core, sepsis can be viewed as a “battle” between the immune system and invading pathogens, with the outcome largely determined by the patient’s immune status. Current studies indicate that MDSCs expand under pathological conditions such as infection, inflammation, trauma, and burns, and their early expansion may play a partially protective role in the host (14). However, it remains unclear whether the accumulation and function of MDSCs and their subsets differ between septic and non-septic patients.

To address these questions, we conducted this study to elucidate the differential changes in MDSCs during the early stages of sepsis compared to non-septic conditions, thereby providing evidence to inform clinical decision-making.

## Methods

### Study design and setting

This prospective observational cohort study was conducted at the intensive care unit (ICU) of Hebei General Hospital, a tertiary academic medical center. The protocol was prospectively registered with the Chinese Clinical Trial Registry (Registration ID: ChiCTR2300079024) and received ethical approval from the Institutional Review Board of Hebei General Hospital (Ethical Approval Code: 2023–410). The study adhered to the ethical principles outlined in the Declaration of Helsinki. Written informed consent was obtained from all participants or their legal representatives prior to study enrollment.

### Study population

**Figure 1:**
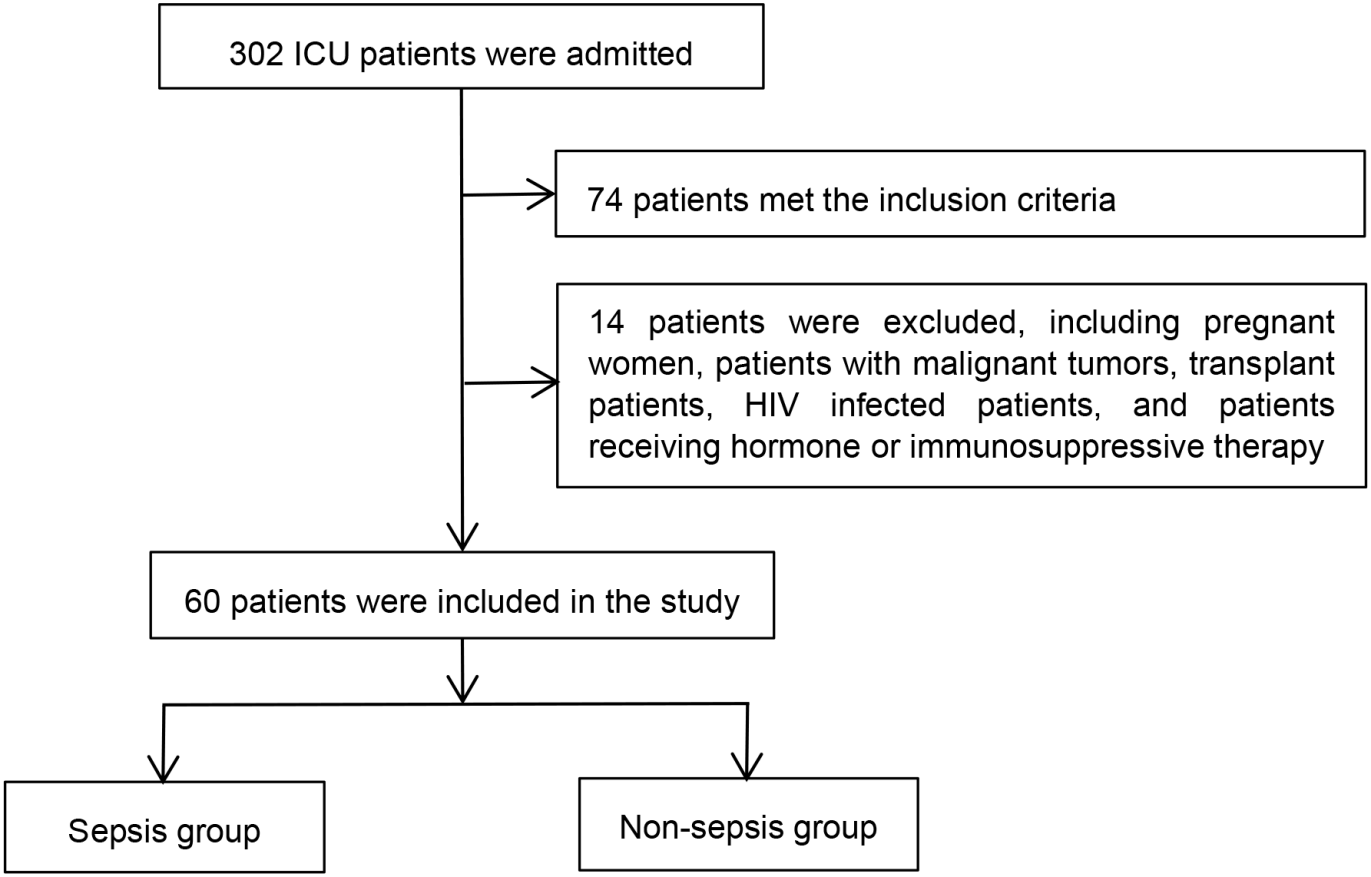
Patients enrolled in our study.

From March to July 2024, critically ill patients meeting the eligibility criteria were enrolled from the intensive care unit (ICU) at Hebei General Hospital and categorized into sepsis and non-sepsis groups (**Figure 1**).

The inclusion criteria for the study were: age ≥ 18 years, APACHE II score ≥ 15, onset time ≤ 72 hours, and provision of informed consent to participate.

Exclusion criteria included pregnancy, malignancy, organ transplantation, HIV infection, and receipt of corticosteroid or immunosuppressive therapy.

### Data collection and processing

Peripheral blood samples were collected in EDTA-anticoagulated tubes within 48 hours of ICU admission. A standardized flow cytometry protocol utilizing a BD Biosciences FACSCanto II flow cytometer with fluorescence-labeled antibodies was employed to quantify circulating CD11b^+^CD33^+^HLA-DR^−^/low cells, which we refer to as MDSC-like cells in this study due to their phenotypic similarity to defined MDSCs. Their subsets were identified as polymorphonuclear MDSC-like cells (PMN-MDSC-like: CD14^−^CD15^+^) and monocytic MDSC-like cells (M-MDSC-like: CD14^+^CD15^−^), as well as lymphocyte subsets and regulatory T cells (Tregs). The laboratory analysis conducted simultaneously includes arginase-1 (ARG-1), inducible nitric oxide synthase (iNOS), complete blood count, serum lactate, interleukin-6 (IL-6), C-reactive protein (CRP), procalcitonin, liver and kidney function, etc. Patients were prospectively followed for 28 days, and the 28-day mortality rate was recorded.

### Flow cytometric analysis of myeloid cells with MDSC phenotypes

#### Peripheral blood mononuclear cell isolation

1. Density gradient preparation: A Ficoll-Paque density gradient was established by layering 1.5 mL of FicollPaque™ PLUS (GE Healthcare) in a 15 mL conical tube.
2. Sample preparation: Whole blood was diluted 1:1 with phosphate-buffered saline (PBS) in a separate tube.
3. Gradient centrifugation: The diluted blood sample was carefully layered onto the Ficoll gradient and centrifuged at 400 ×g for 20 min at 20 °C with brake disengaged.
4. PBMC collection: The mononuclear cell layer at the plasma-Ficoll interface was aspirated using a sterile Pasteur pipette.
5. Cell washing: PBMCs were washed twice with PBS (2 mL per wash) through centrifugation at 300 ×g for 5 min, followed by resuspension in 200 μL PBS.

#### Immunostaining protocol

The following fluorescently conjugated anti-human monoclonal antibodies were titrated in a polystyrene tube:CD15- FITC (clone H198), CD11b-PE (clone ICRF44), CD45-PerCP (clone 2D1), CD33-PE-Cy7 (clone WM55), HLA-DRAPC (clone LN3), CD14-APC-Cy7 (clone 61D3).

#### Staining procedure

100 μL PBMC suspension (1×10^6 cells) was added to the antibody mixture. Vortexed gently and incubated protected from light for 15 min at 4 °C. Washed with 2 mL PBS (300 ×g, 5 min). Resuspended in 500 μL PBS for acquisition.

#### Flow cytometric acquisition and analysis

**Figure 2:**
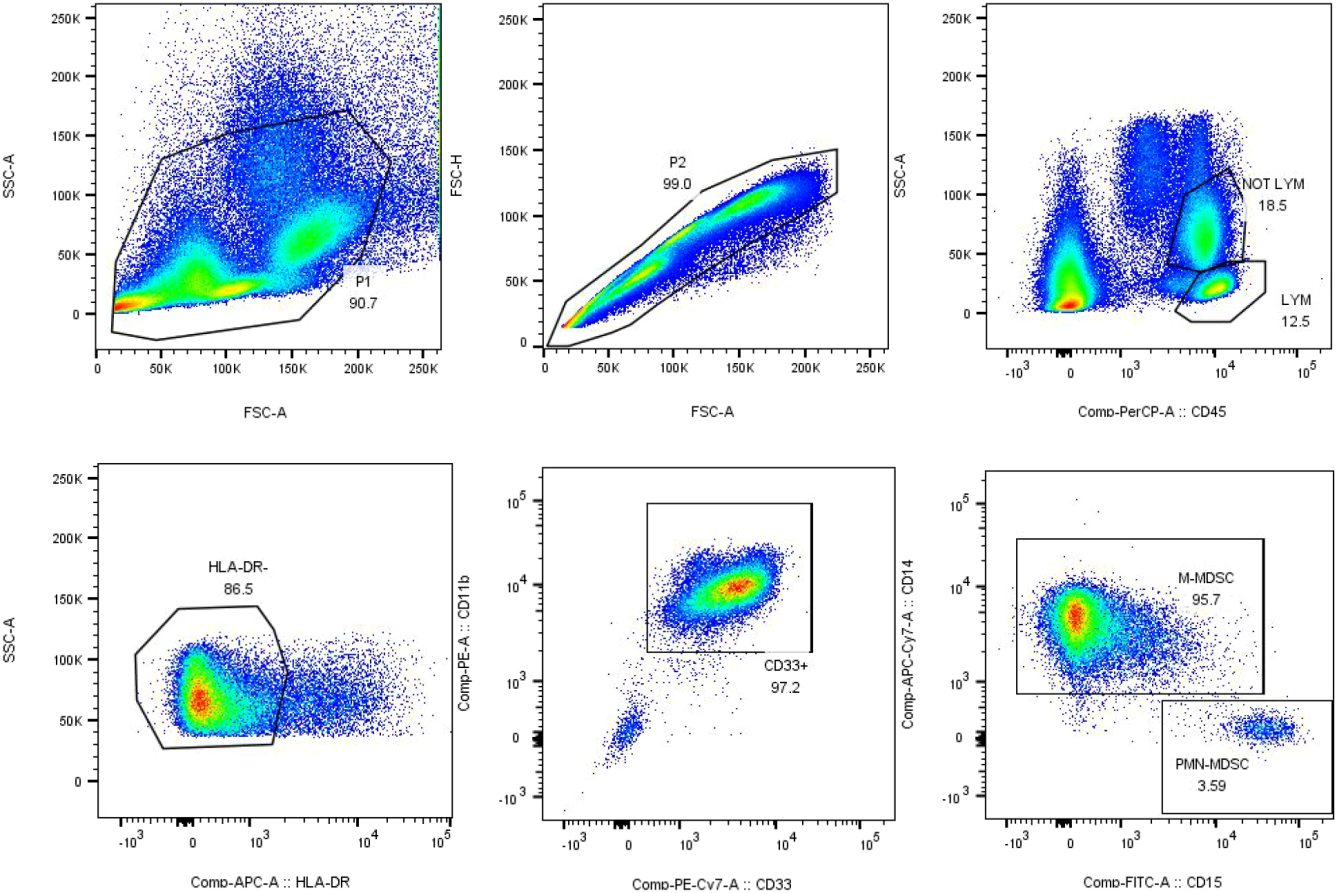
Gated strategy for the identification of MDSC-like cells in critically ill patients.

Samples were analyzed within 2 hours using a BD FACSCanto II flow cytometer (BD Biosciences) with FACSDiva™ software (v8.0.1). The gating strategy (**Figure 2**) was established as follows:

M-MDSCs: CD45^+^ CD33^+^ CD11b^+^ CD14^+^ CD15^−^ HLA-DR^−^

PMN-MDSCs: CD45^+^ CD33^+^ CD11b^+^ CD14^−^ CD15^+^ HLA-DR^−^


$$\text{M}-\text{MDSCs}-\text{like cells}\%=\frac{M-MDSC-like\;cell\;count}{CD45+cell\;count}\times 100\%$$

$$\text{PMN}-\text{MDSCs}-\text{like cells}\%=\frac{\text{PMN}-\text{MDSC}-\text{like cell count}}{\text{CD}45+\text{cell count}}\times 100\%$$

### Flow cytometric analysis of lymphocyte subsets and regulatory T cells

#### Sample preprocessing

Fresh peripheral blood was gently mixed by inversion, and 30–50 μL of the blood was aliquoted and incubated with fluorescence-conjugated antibodies in a light-protected environment for 15 minutes. Hemolysin was then added to lyse red blood cells, followed by washing steps to clarify the cells before loading onto the flow cytometer for analysis.

#### Gating strategy

Based on the principles of flow cytometry, the primary cell population was gated using forward scatter (FSC, reflecting relative cell size) and side scatter (SSC, indicating internal structural complexity). Subsequently, lymphocytes were further gated using CD45 expression combined with SSC. Cell subsets were then identified based on surface-specific antigen expression:

T lymphocytes: CD3+

Helper/inducer T lymphocytes: CD3+ CD4+

Suppressor/cytotoxic T lymphocytes: CD3+ CD8+

B lymphocytes: CD3− CD19+

NK lymphocytes: CD3− CD16+ CD56+

Regulatory T cells (Treg): CD3+ CD4+ CD25+ CD127low/−

#### ARG-1, iNOS, interleukin-6 and interleukin-10

Principle: The enzyme-linked immunosorbent assay (ELISA) was based on antigen-antibody binding for quantification.

All other indicators were completed by the hospital laboratory department: complete blood count (CBC), oxygenation index and lactate, C-reactive protein (CRP), procalcitonin (PCT), coagulation function (prothrombin time [PT] and activated partial thromboplastin time [APTT]), liver function (alanine aminotransferase [ALT] and albumin [ALB]), creatinine, B-type natriuretic peptide (BNP), creatine kinase-MB (CK-MB), and cardiac troponin.

#### Statistical analysis

Statistical analyses were performed using SPSS 25.0 software. Data distribution was initially assessed through normality and homogeneity of variance tests. Normally distributed continuous variables were expressed as mean ± standard deviation (SD) and compared using analysis of variance (ANOVA) for intergroup comparisons, while non-normally distributed quantitative data were summarized as median with interquartile range (IQR) and analyzed via non-parametric methods (eg, Mann–Whitney U-test). Categorical variables were described as frequency (%) and compared using chi square tests. A two-tailed p-value < 0.05 was considered statistically significant for all analyses.

## Results

### Clinical data

A total of 60 critically ill patients were enrolled in this study and divided into a sepsis group (n=38) and a non-sepsis group (n=22). The non-sepsis group included patients with conditions such as trauma, traumatic brain injury, acute pancreatitis, and stroke (**Table 1**). There were no significant differences between the two groups in terms of gender, age, APACHE II score, length of ICU stay, hospital stay, or 28-day mortality. However, the Charlson Comorbidity Index (CCI) was significantly higher in the sepsis group compared to the non-sepsis group [(4.000 ± 1.801) *vs*. (2.180 ± 1.615), P = 0.005].

**Table 1:** Characteristics of patients in the sepsis and non-sepsis groups.

Characteristic	Sepsis group	Non-sepsis group	χ2/t/Z	P value
Number of patients	38	22	–	–
Gender, male	28	15	0.208	0.649
Age (years)	67.870 ± 11.283	61.180 ± 16.005	1.892	0.063
Charlson omorbidity index(CCI)	4.000 ± 1.801	2.180 ± 1.615	2.919	**0.005**
APACHE II score	23.760 ± 7.981	20.910 ± 5.227	1.499	0.139
Length of ICU stay(days)	5.271(2.604,11.438)	7.042(3.415,12.344)	-0.652	0.514
Length of hospital stay(days)	15.230(10.833,24.677)	14.208(8.031,23.240)	-0.483	0.629
28 day mortality rate	13(34.211%)	5(22.727%)	0.875	0.350

p values < 0.05 are highlighted in bold.

### Differences in the frequency of MDSC-like cells and levels of metabolites between sepsis and non-sepsis groups

The percentage of total MDSC-like cells and M-MDSC-like cells was lower in the sepsis group than in the non-sepsis group, with median values [interquartile range] of 4.180% (1.590, 7.281) *vs*. 9.821% (3.371, 17.156) (P = 0.006) and 3.173% (1.480, 5.834) *vs*. 9.529% (3.322, 16.856) (P = 0.003), respectively. In contrast, no significant difference was observed in the percentage of PMN-MDSC-like cells between the two groups. The level of ARG-1 was significantly higher in the sepsis group compared to the non-sepsis group [8.176 (4.917, 18.507) *vs*. 4.396 (1.389, 9.634), P = 0.030], whereas no difference was detected in iNOS levels. The detailed results are presented in **Table 2**.

**Table 2:** Levels of MDSC-like cell subgroups and metabolites in the sepsis and non-sepsis groups.

Characteristic	Sepsis (n=38)	Non-sepsis (n=22)	t/Z	P value
Total MDSC-like cells (%)	4.180 (1.590,7.281)	9.821 (3.371,17.156)	-2.746	**0.006**
M-MDSC-like cells (%)	3.173 (1.480,5.834)	9.529 (3.322,16.856)	-3.022	**0.003**
PMN-MDSC-like cells (%)	0.066 (0.021,0.237)	0.053 (0.017,0.113)	-1.304	0.192
ARG-1 (ng/ml)	8.176 (4.917,18.507)	4.396 (1.389,9.634)	-2.171	**0.030**
iNOS (pg/ml)	2346.726 (1266.094,6725.879)	2985.794 (1385.209,12016.882)	-1.289	0.198

p values < 0.05 are highlighted in bold.

### Comparison of inflammatory and immune indicators between sepsis and non-sepsis groups

The levels of IL-6, CRP, and PCT were significantly higher in the sepsis group than in the non-sepsis group. The median [interquartile range] IL-6 level was 197.550 (101.785, 438.775) pg/mL versus 71.030 (49.970, 208.100) pg/mL (P = 0.011). CRP levels (mean ± SD) were 173.711 ± 79.566 mg/L compared to 93.080 ± 64.839 mg/L (P<0.001). PCT levels were 8.835 (1.920, 24.433) ng/mL versus 0.720 (0.298, 2.435) ng/mL (P<0.001).Additionally, lymphocyte counts were significantly lower in the sepsis group [0.610 (0.485, 1.203) ×10^9^/L *vs*. 0.880 (0.780, 1.245) ×10^9^/L, P = 0.017]. No statistically significant differences were observed between the two groups in the levels of IL-10, WBC, neutrophil count, CD4^+^ T cells, CD8^+^ T cells, B cells, NK cells, or Treg cells. The detailed results are presented in **Table 3**.

**Table 3:** Levels of inflammatory and immune indicators in the sepsis and non-sepsis groups.

Characteristic	Sepsis (n=38)	Non-sepsis (n=22)	t/Z	P value
IL-6 (pg/mL)	197.550 (101.785,438.775)	71.030 (49.970,208.100)	-2.531	**0.011**
IL-10 (pg/mL)	3.974 (1.217,9.273)	2.346 (1.250,5.475)	-1.112	0.266
CRP (mg/L)	173.711 ± 79.566	93.080 ± 64.839	4.263	**0.000**
PCT (ng/mL)	8.835 (1.920,24.433)	0.720 (0.298,2.435)	-4.318	**0.000**
WBC (x10^9^/L)	12.195 (7.878,19.050)	10.245 (8.498,15.213)	-0.752	0.452
Neutrophils (x10^9^/L)	10.255 (5.550,16.028)	8.495 (6.895,13.580)	-0.782	0.434
lymphocyte (x10^9^/L)	0.610 (0.485,1.203)	0.880 (0.780,1.245)	-2.386	**0.017**
CD4+ (A/ul)	248.065 (130.750,410.000)	267.000 (219.000,383.250)	-0.805	0.421
CD8+ (A/ul)	159.670 (66.948,229.250)	181.000 (127.750,246.000)	-0.844	0.399
B cell (A/ul)	148.500 (85.000,220.890)	155.500 (103.250,230.250)	-0.721	0.471
NK (A/ul)	78.000 (49.750,178.500)	94.000 (63.250,182.000)	-0.598	0.550
Treg (%)	6.250 (5.100,9.500)	6.850 (5.200,9.925)	-0.514	0.607

p values < 0.05 are highlighted in bold.

### Comparison of organ function indicators between sepsis and non-sepsis groups

Patients in the sepsis group had a significantly higher SOFA score compared to the non-sepsis group [(8.110 ± 3.944) *vs*. (6.410 ± 2.482), P = 0.046]. Statistically significant differences were also observed between the two groups in PT, APTT, ALB, CR, and BNP levels. The median (interquartile range) PT was 14.200 s (13.075, 18.350) *vs*. 12.550 s (12.275, 13.625) (P = 0.006); APTT was 33.650 s (28.350, 38.325) *vs*. 28.000 s (24.775, 32.575) (P = 0.006); mean ± SD ALB was 26.255 ± 6.231 g/L *vs*. 31.459 ± 5.217 g/L (P = 0.002); median CR was 110.300 μmol/L (64.225, 180.825) *vs*. 72.500 μmol/L (59.175, 92.300) (P = 0.018); and median BNP was 1923.500 pg/mL (528.050, 7198.750) *vs*. 359.900 pg/mL (84.305, 1344.650) (P = 0.001).

Although platelet counts and the oxygenation index(OI) were lower in the sepsis group (165.605 ± 77.561 *vs*. 196.000 ± 90.987 ×10^9^/L and 187.184 ± 92.948 *vs*. 213.584 ± 90.570, respectively), the differences were not statistically significant. No significant differences were found between the two groups in ALT, CK-MB, troponin, or lactate levels. The detailed results are presented in **Table 4**.

**Table 4:** Organ function index levels in sepsis and non-sepsis groups.

Characteristic	Sepsis (n=38)	Non-sepsis (n=22)	t/Z	P value
SOFA score	8.110 ± 3.944	6.410 ± 2.482	2.043	**0.046**
PLT (×10^9^/L)	165.605 ± 77.561	196.000 ± 90.987	-1.372	0.175
PT (s)	14.200 (13.075,18.350)	12.550 (12.275,13.625)	-2.762	**0.006**
APTT (s)	33.650 (28.350,38.325)	28.000 (24.775,32.575)	-2.731	**0.006**
ALT (U/L)	17.750 (11.370,39.225)	29.900 (15.850,57.700)	-1.787	0.074
ALB (g/L)	26.255 ± 6.231	31.459 ± 5.217	-3.301	**0.002**
CR (μmol/L)	110.300 (64.225,180.825)	72.500 (59.175,92.300)	-2.362	**0.018**
OI (mmHg)	187.184 ± 92.948	213.584 ± 90.570	-1.070	0.289
BNP (pg/mL)	1923.500 (528.050,7198.750)	359.900 (84.305,1344.650)	-3.237	**0.001**
CK-MB (U/L)	14.400 (10.525,28.975)	13.750 (10.415,27.975)	-0.345	0.730
Troponin (pg/mL)	0.042 (0.023, 0.160)	0.030 (0.011,0.126)	-1.121	0.263
LAC (mmol/L)	2.415 (1.550,5.058)	2.310 (1.750,3.835)	-0.276	0.782

p values < 0.05 are highlighted in bold.

### Differences in the frequency of MDSC-like cell subsets and metabolite levels between survivors and non-survivors in the sepsis group

Patients in the sepsis group were stratified by 28-day survival status into a survival group (n=25) and a non-survival group (n=13). Comparison between survivors and non-survivors showed no statistically significant differences in the percentages of total MDSC-like cells and M-MDSC-like cells, or in the levels of ARG-1 and iNOS. However, the percentage of PMN-MDSC-like cells was significantly higher in non-survivors than in survivors, with median values [interquartile range] of 0.098% (0.028, 0.571) versus 0.064% (0.020, 0.160) (P = 0.012). The detailed results are presented in **Table 5**.

**Table 5:** Comparison of MDSC-like cell subsets and metabolites between survivors and non-survivors in the sepsis group.

Characteristic	Survivors (n=25)	Non-survivors (n=13)	Z	P value
Total MDSC-like cells (%)	4.450 (1.825,7.489)	3.910 (0.810,6.996)	-0.631	0.538
M-MDSC-like cells (%)	4.262 (1.726,7.391)	2.508 (0.741,4.762)	-0.385	0.701
PMN-MDSC-like cells (%)	0.064 (0.020,0.160)	0.098 (0.028,0.571)	-2.508	**0.012**
ARG-1 (ng/ml)	7.602 (4.910,12.875)	11.351 (4.829,24.321)	-0.969	0.332
iNOS (pg/ml)	2299.112 (1150.615,7002.931)	3097.468 (1390.206,8051.330)	-0.354	0.723

p values < 0.05 are highlighted in bold.

## Discussion

Under steady-state conditions, immature myeloid cells, which lack immunosuppressive activity, reside in the bone marrow and are absent from secondary lymphoid organs. However, during pathological changes, the maturation of these cells is blocked. They are released into the peripheral circulation, leading to the expansion of cells with MDSC phenotypes in the body. These immature myeloid cells fail to differentiate normally, arresting at various stages and becoming cells with immunosuppressive functions akin to MDSCs (15). It remains unclear whether the expression of frequency of such MDSC-like cells and their subsets is consistent between septic and non-septic patients.

To investigate the differences in MDSC-like cells during the early stages of disease in septic versus non-septic patients, we specifically enrolled patients with a disease onset time of ≤72 hours and divided them into a sepsis group and a non-sepsis group. Baseline comparison between the two groups showed that the sepsis group had a higher Charlson Comorbidity Index (CCI), indicating that these patients had more pre-existing comorbidities. However, there were no differences between the two groups in terms of gender, age, APACHE II score, duration of ICU treatment, hospital length of stay, or 28-day mortality.

This study found that there are differences in the expansion of peripheral blood MDSC-like cells between septic and non-septic patients in the early stages of the disease: the percentages of both total MDSC-like cells and M-MDSC-like cells were lower in the septic group compared to the non-septic group, while no significant difference was observed in PMN-MDSC-like cells between the two groups. Additionally, lymphocyte counts were decreased in septic patients. The pathological activation of MDSC-like cells in sepsis can be induced by pathogen-associated molecular patterns such as lipopolysaccharide (LPS), cytokines including high mobility group protein B1 (HMGB1), IFN-γ, IL-1β, IL-4, IL-6, IL-7, IL-10, IL-13, TNF, and CXCL3, as well as acute-phase proteins like α2-macroglobulin and serum amyloid A (16). This process is driven by signaling pathways such as STAT3, which promotes the massive generation of these cells (17). Their potent immunosuppressive function is linked to the reinforcement and stabilization of pathways like p38-MAPK and long non-coding RNAs (e.g., Hotairm1) (18, 19), accompanied by persistent metabolic reprogramming, including mitochondrial dysfunction (20). These factors collectively contribute to a profound and prolonged state of immunosuppression, which is closely associated with poor clinical outcomes.

During sepsis, myeloid progenitor cells expand and shift toward myeloid differentiation, leading to increased production of neutrophils and monocytes, which enhances the innate immune response against infection (21). Conversely, lymphocytes undergo significant depletion and apoptosis (22), and lymphopoiesis is impaired (21), resulting in decreased lymphocyte counts. This imbalance between myelopoiesis and lymphopoiesis reflects the immune system’s priority in generating cells that can rapidly respond to infection. Additionally, the systemic energy metabolism disorder, tissue hypoxia, and microcirculatory dysfunction induced by sepsis further compromise the maintenance of immunometabolism (23).

In contrast to sepsis, the expansion of MDSC-like cells following trauma is associated with the activation of acute stress axes, such as the hypothalamic-pituitary-adrenal axis, and the release of damage-associated molecular patterns (DAMPs) (24). Zhang et al. (25) confirmed that hormone administration alone can promote MDSC-like cell expansion. Studies have shown that post-traumatic MDSC-like cells modulate the Treg/Th17 and Th2/Th1 balance via LOX1 markers, NF-κB, and TGF-β1 signaling pathways, exerting anti-inflammatory effects and correlating with favorable prognosis (26). Hosomi et al. (27) found that PMN-MDSC-like cells can infiltrate the injury site early after traumatic brain injury. Following cerebral infarction, nociceptive neurons in the bone marrow release calcitonin gene-related peptide (CGRP), promoting the proliferation and mobilization of MDSC-like cells and alleviating intracerebral inflammation (28). In acute pancreatitis, substances released by pancreatic cells activate pattern recognition receptors and drive the generation and release of MDSC-like cells from the bone marrow through cytokines such as GM-CSF, G-CSF, and IL-6 (29). Based on the above, we propose that the differences in early MDSC-like cell expansion between the sepsis and non-sepsis groups may be related to factors such as distinct immune mobilization patterns and metabolic environments.

The function of canonical MDSCs largely relies on arginase (ARG-1) and inducible nitric oxide synthase (iNOS). Both enzymes share the substrate L-arginine, which is metabolized into urea/L-ornithine and nitric oxide/L-citrulline, respectively (30). High expression of ARG-1 in MDSCs can deplete L-arginine in the microenvironment, thereby inhibiting T-cell activation. Increased iNOS expression, on the other hand, directly suppresses and induces apoptosis in T cells via nitric oxide (NO) (31). PMN-MDSCs express IL-10 and IL-12, while M-MDSCs express IL-6, IL-10, and TGF-β. Both subsets produce significant levels of arginase, whereas iNOS is primarily derived from M-MDSCs (32). Interestingly, this study found that although the percentage of MDSC-like cells in the peripheral blood of the sepsis group was lower than that in the non-sepsis group, the ARG-1 level was higher in the sepsis group, with no significant difference in iNOS between the groups. This discrepancy may be attributed to the sepsis environment being more conducive to inducing ARG-1 expression in these cells (33), and MDSC-like cells can migrate to organs such as the spleen and liver or to sites of infection to exert their functions (34, 35). Additionally, MDSC-like cells may exhibit reduced survival due to mitochondrial dysfunction, and in sepsis, local tissue neutrophils or macrophages might also highly express ARG-1 (36, 37). These factors could explain the inconsistency between peripheral blood MDSC-like cell counts and ARG-1 levels, though further validation is required.

MDSCs are regarded as a “double-edged sword” (38), and their early expansion may exert protective effects. For instance, in fungal infections, MDSCs can alleviate inflammatory damage by suppressing T-cell activity but may also facilitate fungal immune escape (39). *In vitro*, prostaglandin E2 (PGE2)-induced MDSCs can modulate immune cell infiltration, protect the intestinal barrier, and ameliorate experimental autoimmune encephalomyelitis (40). In the early phase of ischemic stroke, MDSCs contribute to the improvement of cerebral ischemic injury (41); similarly, in a rat model of bone trauma, MDSC proliferation promotes fracture healing (42). In this study, neither patient group showed a significant elevation in IL-10 during the early stage, which may indicate that the classical anti-inflammatory response was not predominant at the sampling time point. However, this does not exclude the concurrent presence of other immunosuppressive mechanisms, such as lymphocyte apoptosis and exhaustion, as well as MDSC-like cell-mediated suppression via pathways involving ARG-1/iNOS.

The immune status in sepsis undergoes dynamic changes, involving complex interactions among multiple immune cells and molecules (43). Pathogen- or damage-associated molecular patterns activate innate immunity through pattern recognition receptors, triggering a massive release of cytokines and chemokines and activating the coagulation and complement systems. Concurrently, a reduction in the number of immune effector cells leads to exacerbated immunosuppression and infection (44, 45), and may even provoke a cytokine storm and multiple organ dysfunction syndrome (MODS) (46). This study demonstrated that the sepsis group had significantly higher levels of IL-6, CRP, PCT, and SOFA scores compared to the non-sepsis group, along with elevated PT, APTT, CR, and BNP, as well as reduced ALB. These findings align with the tendency of sepsis to induce organ dysfunction and a hypercatabolic state (24).

Sepsis can impair the function of almost all immune cells, including macrophages, neutrophils, lymphocytes, natural killer (NK) cells, and innate lymphoid cells (ILCs). Such dysfunction may exacerbate immunosuppression, forming a positive feedback loop (47) and ultimately leading to immune paralysis. In this study, although the numbers of CD4+ T cells, CD8+ T cells, B cells, and NK cells in the sepsis group did not show statistically significant differences compared to the non-sepsis group, they exhibited a decreasing trend. Further studies with larger sample sizes are required to clarify the roles of these lymphocyte subsets.

To further analyze the significance of MDSC-like cell subsets in sepsis, we stratified septic patients by 28-day prognosis into a survival group (n=25) and a non-survival group (n=13). The results showed no significant differences in the percentages of total MDSC-like cells or M-MDSC-like cells between the two groups. However, the percentage of PMN-MDSC-like cells was significantly higher in the non-survival group, suggesting an association between elevated PMN-MDSC-like cells% and poor prognosis in sepsis. Additionally, levels of ARG-1 and iNOS in the non-survival group were numerically higher, though these differences did not reach statistical significance.

### Limitations

This study is a single-center exploratory investigation with several limitations. First, it primarily focused on patients within 72 hours of disease onset and did not dynamically observe changes in MDSC-like cells across different disease stages. Second, the sepsis group included patients with septic shock, while the non-sepsis group comprised various conditions such as trauma, traumatic brain injury, acute pancreatitis, and stroke. Due to the limited sample size, subgroup analyses were not performed. Additionally, blood samples were collected within 48 hours after enrollment, with sampling time points not fully standardized, and some patients had already received clinical interventions prior to blood collection.

The most important limitation is the lack of direct functional validation of identified cell populations. In the absence of *in vitro* inhibition assays, it is impossible to clearly confirm that cells defined by phenotype have complete immunosuppressive ability. Therefore, we refer to these cells as’ MDSC like cells’ to accurately reflect their phenotypic characteristics while acknowledging the uncertainty of this function. Relying solely on changes in cell frequency and ARG-1 expression levels is not sufficient to fully confirm alterations in its immunosuppressive activity.

Methodologically, this study employed a PBMC-based isolation protocol, primarily because it is currently the most mature and widely used standard method in MDSC research, facilitating direct comparison with a substantial body of existing literature. This approach effectively enriches monocytes while removing interference from granulocytes and red blood cells. It is particularly suitable for the analysis of M-MDSC-like cells and provides a stable cell count foundation for standardized immunostaining. However, standard Ficoll density gradient centrifugation leads to the loss of granulocytes. Therefore, the PMN-MDSC-like cells identified in this study (i.e., CD15^+^ cells within the PBMC fraction) likely represent only a low-density subset, and our data may not fully reflect the absolute abundance of all cells with a PMN-MDSC phenotype in whole blood.

Nevertheless, the observed changes in MDSC-like cell frequency and ARG-1 levels in this study still hold significant indicative value and provide direction for future research.

## Conclusions

In the early stage of sepsis, patients exhibited lower percentages of total MDSC-like cells and M-MDSC-like cells, along with reduced lymphocyte counts, compared to the non-sepsis group. However, their ARG-1 levels were significantly higher. Patients with sepsis also had higher SOFA scores, indicating a greater susceptibility to multi-organ dysfunction. Furthermore, an elevated percentage of PMN-MDSC-like cells was associated with poorer prognosis in septic patients.

## Data Availability

The datasets presented in this article are not readily available because the datasets generated and analyzed during this study contain confidential patient information and are not publicly available due to ethical restrictions and privacy regulations imposed. De-identified data can be made available to qualified researchers upon reasonable request, subject to a formal data sharing agreement. Requests to access the datasets should be directed to Jia lijing, jlj011365@163.com. Data for this study are publicly available in Zenodo, DOI: 10.5072/zenodo.473873.
